# Clonal and plasmidic dissemination of critical antimicrobial resistance genes through clinically relevant ExPEC and APEC-like lineages (ST) in the dairy cattle population of Québec, Canada

**DOI:** 10.3389/fmicb.2023.1304678

**Published:** 2024-01-18

**Authors:** Maud de Lagarde, John Morris Fairbrother, Marie Archambault, Simon Dufour, David Francoz, Jonathan Massé, Hélène Lardé, Cécile Aenishaenslin, Marie-Eve Paradis, Yves Terrat, Jean-Philippe Roy

**Affiliations:** ^1^Department of Clinical Sciences, Faculty of Veterinary Medicine, Université de Montréal, Saint-Hyacinthe, QC, Canada; ^2^Regroupement Front de Recherche du Québec – Nature et Technologie (FRQNT) Op+lait, Saint-Hyacinthe, QC, Canada; ^3^World Organization of Animal Health Reference Laboratory for Escherichia coli, Faculty of Veterinary Medicine, Université de Montréal, Saint-Hyacinthe, QC, Canada; ^4^Swine and Poultry Infectious Diseases Research Center (CRIPA-FQRNT), Faculty of Veterinary Medicine, Université de Montréal, Saint-Hyacinthe, QC, Canada; ^5^Department of Pathology and Microbiology, Faculty of Veterinary Medicine, Université de Montréal, Saint-Hyacinthe, QC, Canada; ^6^Department of Clinical Sciences, Ross University School of Veterinary Medicine, St. Kitts, St. Kitts and Nevis; ^7^Groupe de Recherche en Épidémiologie des Zoonoses et Santé Publique, Faculté de Médecine Vétérinaire, Université de Montréal, Saint-Hyacinthe, QC, Canada; ^8^Centre de recherche en santé publique de l’Université de Montréal et du Centre Intégré Universitaire de Santé et de Service Sociaux (CIUSSS) du Centre-Sud-de-l’Île-de-Montréal, Montréal, QC, Canada; ^9^Association des médecins vétérinaires praticiens du Québec, Saint-Hyacinthe, QC, Canada; ^10^Consortium Santé Numérique de l’Université de Montréal, Montréal, QC, Canada

**Keywords:** *Escherichia coli*, gene spread, manure pit, legislation, calf

## Abstract

Antimicrobial resistance can be effectively limited by improving the judicious use of antimicrobials in food production. However, its effect on the spread of AMR genes in animal populations is not well described. In the province of Québec, Canada, a new legislation implemented in 2019 has led to an unprecedented reduction in the use of critical antimicrobials in dairy production. We aimed to investigate the potential link between ESBL/AmpC *E. coli* isolated before and after legislation and to determine the presence of plasmids carrying genes responsible for critical AMR. We collected fecal samples from calves, cows, and manure pit from 87 Québec dairy farms approximately 2 years before and 2 years after the legislation came into effect. The whole genomes of 183 presumptive ESBL/AmpC *E. coli* isolated after cefotaxime enrichment were sequenced. Their phylogenetic characteristics (MLST, serogroup, cgMLST) and the presence of virulence and resistance genes and replicons were examined. A maximum likelihood phylogenetic tree was constructed based on single nucleotide polymorphism (SNPs). We identified 10 clonal lineages (same cgMLST) and 7 clones (SNPs ≤ 52). Isolates belonging to these clones could be found on different farms before and after the legislation, strongly suggesting a clonal spread of AMR genes in the population during this 4-year period. All isolates were multidrug resistant (MDR), with clone 2 being notable for the presence of macrolide, fluoroquinolone, and third-generation cephalosporin resistance genes. We also identified clinically relevant ExPEC (ST10) and APEC-like lineages (ST117, ST58, ST88) associated with the presence of ExPEC and APEC virulence genes, respectively. Our data also suggests the presence of one epidemic plasmid belonging to the IncY incompatibility group and carrying *qnrs1* and *bla_*CTX–M–*15_*. We demonstrated that AMR genes spread through farms and can persist over a 4-year period in the dairy cattle population through both plasmids and *E. coli* clones, despite the restriction of critical antimicrobial use. MDR ExPEC and APEC-like STs are present in the normal microbiota of cattle (more frequently in calves). These data increase our knowledge on gene dissemination dynamics and highlight the fact that biosecurity measures should be enhanced in this industry to limit such dissemination.

## 1 Introduction

The global burden attributable to bacterial antimicrobial resistance (AMR) has been estimated at 1.27 million human deaths in 2019 (Antimicrobial Resistance Collaborators, 2022). Therefore, tackling AMR has become a public health priority worldwide. In recent years, several international organizations [World Health Organization (WHO), Food and Agriculture Organization (FAO), World Organization for Animal Health (WOAH)] and several countries have developed strategies to fight AMR (United Nation General Assembly, 2016). Improving the judicious usage of antimicrobials (AM) in food production is one of them (Tang et al., 2017). The province of Québec (Canada) adopted a new legislation in February 2019, to limit usage of category I AMs (e.g., third generation cephalosporins, fluoroquinolones or polymyxin B) of the Health Canada classification (Government of Canada, 2009) in production animals (Roy et al., 2020). This new regulation has been very effective in reducing the use of these AMs (Millar et al., 2022). However, the effect of modification of antimicrobial use in food-producing animals on AMR gene dissemination in animal populations is not well described. It should be noted, however, that WHO and Health Canada categorizations are similar but exhibit some differences (Lardé et al., 2021). For example, Category I AM, as defined by Health Canada, aligns with the highest-priority critically important antimicrobials (HPCIA) outlined by the WHO, except for macrolides, which the WHO categorizes as HPCIA whereas they are classified as Category II AM in Canada.

*Escherichia coli* is a ubiquitous Gram-negative rod. It is mostly commensal and can be found in the gut of all mammals (Gyles et al., 2010). However, depending on the presence of specific virulence and/or resistance genes, it can also cause multiple diseases (from mild diarrhea to fatal sepsis) in both humans and animals. High risk clones were described within the last decades (Mathers et al., 2015; De Lagarde et al., 2021). They are defined as emergent, multi-drug resistant (MDR), highly pathogenic and capable of potent dissemination (De Lagarde et al., 2021), and they are recognized as a cause of major disease outbreaks worldwide (Kocsis et al., 2022). Successful dissemination of these drug-resistant and pathogenic *E. coli* depends on the acquisition and carriage of niche-specific characteristics that allow for steady colonization and persistence. A critical evolutionary step in the emergence of these MDR clones, is the acquisition of multi-drug resistance and/or fitness genes through plasmids. The study of the emergence of these clones and the diverse plasmids carrying resistance and virulence genes present in the animal population is essential to develop fighting strategies such as vaccines and limit the spread through improvement of biosecurity measures.

Extraintestinal pathogenic *E. coli* (ExPEC) are responsible for a significant number of human infections worldwide (Poolman and Wacker, 2015). These strains typically reside in the intestinal microbiota, and from there, they emerge to cause infections outside the intestines. A few specific lineages of ExPEC classified with their sequence type (ST) are responsible for many of these infections. The top five clinically relevant STs are ST131, ST69, ST10, ST405, and ST38 (Manges et al., 2019). Certain sets of ExPEC are causing specific colibacilloses in poultry and have been therefore designed as avian pathogenic *E. coli* (APEC). The APEC predominant lineages are ST131, ST117, ST23, ST428, ST355 (Johnson et al., 2022). Interestingly, the ST131 lineage can be of importance in both poultry and humans. These ExPEC-APEC strains are also known for their association with the acquisition of new and concerning AMR genes. The clinical and economic impact of ExPEC infections, as well as their optimal management in the face of increasing AMR, pose significant challenges that are not fully recognized. Understanding the genetic factors that contribute to the persistence, predominance, and competitiveness of ExPEC strains within the gut microbiota remains unclear but may provide insights into the success of these lineages. Cattle are not recognized as reservoir for ExPEC, however, there are recent evidence that this information might need to be revised (Salaheen et al., 2023).

Our research team already established the portrait of both antimicrobial usage (AMU) and AMR in dairy farms in Québec prior to the regulation implementation (Lardé et al., 2021; Massé et al., 2021), and the impact that the regulation had on AMU and AMR in *E. coli* isolates (De Lagarde et al., 2022; Millar et al., 2022). We also characterized antimicrobial resistance genes in ESBL/AmpC isolates before the regulation implementation and determined the correlation between phenotypes and genotypes in these isolates (Massé et al., 2023). However, we have not yet characterized the ESBL/AmpC *E. coli* isolated after the restriction of category I AMU, and the persistence of resistance genes, clones, and plasmids. Therefore, the first objective of this study was to characterize and assess the putative phylogenic link between ESBL/AmpC *E. coli* gathered over a 4-year period (pre- and post- regulation implementation). Secondly, we aimed to determine the presence of plasmids carrying genes responsible for resistance to critical AM in these isolates, and whether these plasmids were able to persist over a 4-year period.

## 2 Materials and methods

### 2.1 Selection of herds and sample collection

The herd selection and sample collection were described previously (Massé et al., 2021; De Lagarde et al., 2022). Briefly, we used an observational prospective cohort study on 87 commercial dairy farms. Prior to initiating the research, the protocol was approved by the Animal Use Ethics and the Research Ethics Committees of the Université de Montréal (20-Rech-2085). The 87 farms were in Montérégie, Centre-du-Québec and Estrie, Québec, Canada, three of the main dairy areas of Québec. These regions were selected based on the proximity to the veterinary faculty of the Université de Montréal. The farms were randomly selected in the three regions from a list of dairy farms provided by the Ministère de l’Agriculture, des Pêcheries et de l’Alimentation du Québec (MAPAQ; Québec’s department of agriculture, fisheries, and food). In total, four samplings were carried out. The two first samplings were performed approximately 2 years before the regulation implementation (April to June 2017, October to November 2017). Two additional samplings were performed approximately 2 years after the regulation implementation (August to September 2020 and February to March 2021). The timeline of the sampling was previously illustrated (De Lagarde et al., 2022).

The sampling protocol was previously described (De Lagarde et al., 2022). Briefly, on each visit, fecal samples were collected from five pre-weaned calves and mixed to obtain a composite sample. Fecal samples of five lactating cows were also collected and mixed to obtain another composite sample. On each farm, a convenience sample was assembled based on accessibility of the calves and cows. Fecal samples were obtained directly from the rectum for calves and freshly voided cow feces were obtained from the floor. A composite manure sample was also collected from two convenient locations in the manure pit. For each composite sample, approximately 25 g of feces or manure were placed in a 50 mL sterile tube and stored immediately on ice at the farm. Samples were processed in the laboratory within < 24 h. A preservative medium (peptone water with 30% glycerol) was added to the sample at a 1:1 volume to weight ratio; samples were then homogenized and frozen at −80°C.

As part of a wider project on AMR and AMU, we also gathered information on farm location and veterinarian care.

### 2.2 Bacterial isolation and presumptive ESBL/AmpC *E. coli* identification

The protocol for bacterial isolation was previously described (Massé et al., 2021). Briefly, composite fecal samples were processed according to the laboratory protocol of the European Union Reference Laboratory on Antimicrobial Resistance for the recovery of ESBL-, AmpC- and carbapenemase-producing *E. coli* from composite fecal samples. The protocol is available online at https://www.eurl-ar.eu/protocols.aspx. One gram of each composite fecal or manure sample was added to 9 mL of Buffered Peptone Water, then incubated at 37°C for 20 h. One loop (10 μl) was streaked onto a MacConkey agar plate containing 1 mg mL-1 of cefotaxime, then incubated at 44°C for 20 h. Lactose positive colonies were subcultured onto Columbia agar with 5% sheep blood, and then incubated overnight at 37°C. Identification of *E. coli* was confirmed by MALDI-TOF MS. Composite samples with at least one *E. coli* colony isolated with this technique were labeled as presumptive ESBL/AmpC *E. coli*. All *E. coli* selected were incubated for 24 h at 37°C in Luria-Bertani (LB) broth then mixed 50:50 with 30% glycerol and stored at −80°C.

### 2.3 DNA extraction, library preparation and whole genome sequencing

Due to financial and logistic restrictions, we sequenced 183 isolates in total. Most isolates were randomly selected within the collection pre- and post- regulation (178/183), ensuring that we had a similar number of isolates for both periods. Additionally, a subset of 5 isolates in the pre-regulation collection were selected because they presented an atypical phenotype (Massé et al., 2023). Genomic DNA was extracted using QIAamp DNA Mini Kit for DNA following manufacturer’s guidelines (Qiagen, Hilden, Germany). The isolates gathered before the regulation were sequenced with MiSeq platform with 2 × 300 paired end runs after library preparation with the Illumina Nextera XT DNA Library preparation kit, according to the manufacturer’s instructions. The isolates gathered after the regulation were sequenced on the Illumina (San Diego, CA) iSeq100 platform with 2 × 150 paired end runs after library preparation with the Illumina DNA prep kit (former Nextera Flex kit), according to the manufacturer’s instructions.

Illumina platform was used to assemble genomes using SPADES 3.9.0 (Bankevich et al., 2012). An assembly was rejected if the number of contigs (> 500 bp) was > 400 or if the N50 was < 50,000. Details of data assembly quality are available in Supplementary Table 1.

### 2.4 Multi locus sequence typing (MLST), serotype, phylogroup and adhesin *fimH*

Multi locus sequence typing (MLST) (Larsen et al., 2012) (minimal depth for the detection of an MLST allele was 5x), O and H serotypes (Joensen et al., 2015) (85% identity and 60% coverage to count as a hit) and the *fimH* subtype (Roer et al., 2017) (95% identity to count as a hit) were determined by analysis of generated FASTA files using the Center of Genomic Epidemiology (CGE) platform.^1^ The *fimH* gene is part of the *fim* operon, which encodes for type 1 fimbriae found in most *E. coli* strains.

Phylogroups were determined with *in silico* PCR using the Clermont Typing platform^2^ (Beghain et al., 2018). These parameters (MLST, serotype, phylogroup and *fimH* subtype) will be referred to as phylogenetic characteristics.

### 2.5 Virulence and resistance genes and replicons

To determine the presence of virulence genes, AMR genes and point mutations, Virulence finder, Res Finder 4.0 and Point Finder were used on the CGE platform (Bortolaia et al., 2020). The default parameters were used for each application (90% identity and 60% coverage).

PlasmidFinder (Carattoli et al., 2014) was used to determine the presence of replicons (95% identity and 60% coverage). Mobile genetic element (MGE) (Johansson et al., 2020) was used to identify mobile genetic elements and their relation to antimicrobial resistance genes and virulence factors. The CGE platform was used for both tools.

### 2.6 Phylogenetic analysis

Phylogenetic analysis was performed with an “in-house” pipeline. We used the Digital Research Alliance of Canada computing servers (alliancecan.ca). Raw data preprocessing and phylogenetic analysis were performed as follow: first, we trimmed low quality reads using Trimmomatic (Bolger et al., 2014) with default parameters. Second, trimmed reads were mapped on the reference genome (isolate *E. coli* K12, NC 000913) using BWA (Li and Durbin, 2009). Mapping files were further converted from bam to vcf format and were filtered out using vcftools (Danecek et al., 2011). Finally, Fasttree2 (Price et al., 2010) was used to generate the selection of best models and phylogenetical analyses. Conversion of multiple formats between these different steps has been performed using a combination of freebayes (Garrison and Marth, 2012), samtools (Danecek et al., 2021), gatk software (Van Der Auwera and O’Connor, 2020) and Fasta2Phylip perl program.^3^ The complete python pipeline is available at https://github.com/yterrat/AMR_FMV/blob/main/pipeline_mapping.py.

The SNP phylogenies were annotated with the relevant metadata using iTOL^4^ (Letunic and Bork, 2016).

A clonal lineage was defined as a group of isolates that belong to the same core genome MLST (cgMLST). We used the cgMLSTfinder 1.2 application (Clausen et al., 2018; Zhou et al., 2020) available on CGE platform.

Clones were defined as previously described (De Lagarde et al., 2021). Briefly, only branches from nodes with a bootstrap value of 1 and groups of three or more isolates were considered. Furthermore, the maximum number of SNPs between pairs of isolates within a group, defined as the SNP_*max*_ was < M × T × P where M is the mutation rate of *E. coli*, which has been described as 3 × 10^–6^ per year per site (Grad et al., 2012), T is the number of years between two isolates and P is the total number of nucleotide sites analyzed in all genomes for each isolate.

Singletons were defined as unique isolates in terms of phylogenetic characteristics (MLST, serotype, *fimH* gene and phylogroup).

Each farm where an ESBL/AmpC isolate was retrieved, was geolocated at the centroid of the 3-digit postal code area with GeoPinpointTM Suite (DMTI Spatial Inc.). Then geographical distribution of farms for clones was performed in ArcGIS (version 10.8.1).

## 3 Results

### 3.1 *E. coli* collection description

During the sampling prior to the regulation implementation, 599 fecal samples were collected from 101 dairy farms. A total of 214 ESBL/AmpC-producing *E. coli* were obtained using the selective protocol.

All farmers were contacted in July 2020 and asked to participate in the post-regulation sampling. A total of fourteen farmers either refused or were not able to join the second part of the study. Among the 516 fecal composite samples collected from 87 dairy farms, we recovered 162 presumptive ESBL/AmpC *E. coli*.

The genomes of 182 isolates belonging to the pre- and post-regulation collections (91 in each collection) were completely sequenced. As already published, 85 and 82% of herds were positive for presumptive ESBL/AmpC-producing *E. coli* in at least one sample in 2017 (Massé et al., 2021) and 2020–2021 (De Lagarde et al., 2022), respectively, with no significant differences detected between the two periods (De Lagarde et al., 2022).

### 3.2 Identification of 6 clones over the 4-year period

**FIGURE 1:**
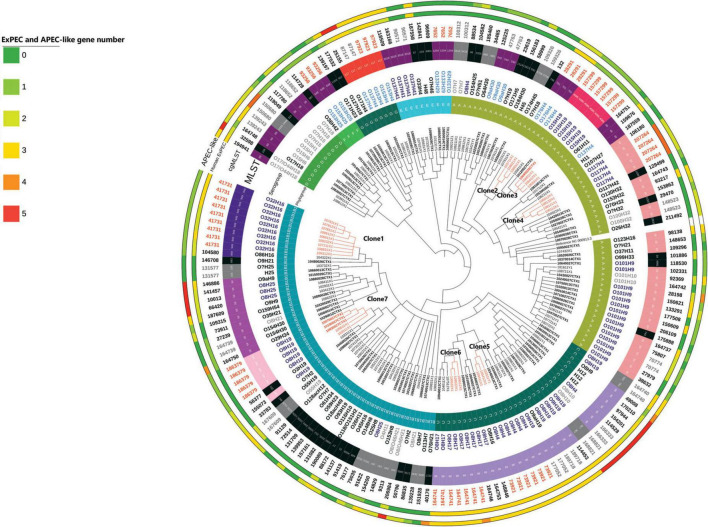
Overall phylogeny based on SNP distance of 182 ESBL/AmpC *E. coli* isolates. The length of the branches is not proportional to the phylogenetic distance. Each branch with a bootstrap value under 1 was collapsed. Samples with ID labels in bold indicate those collected prior to the implementation of the regulation. Isolates are considered as clones if there are not different one from another with more than 52 SNPs. The presence of the 5 APEC-like and 5 human ExPEC predictor genes (Johnson et al., 2003, 2008) is codified by color (from 0 to 5 corresponding to green to red).

Overall phylogeny is presented in Figure 1. Among the 182 isolates, the predominant phylogroups were A (*n* = 61) and B1 (*n* = 57). The phylogroups C, D, E, F and G were also represented. Various serogroups were identified, the most predominant serogroup being O101:H9 (*n* = 15). Numerous MLST were also determined, with the predominant one being the ST10 (*n* = 28). More than 30 types of *fimH* were identified (data not shown on Figure 1). The most predominant was *fimH*54 (*n* = 28), however, it was not possible to associate any of them specifically with any other phylogenetic characteristics. Phylogenetic characteristics of all isolates are also available in Supplementary Table 1.

**FIGURE 2:**
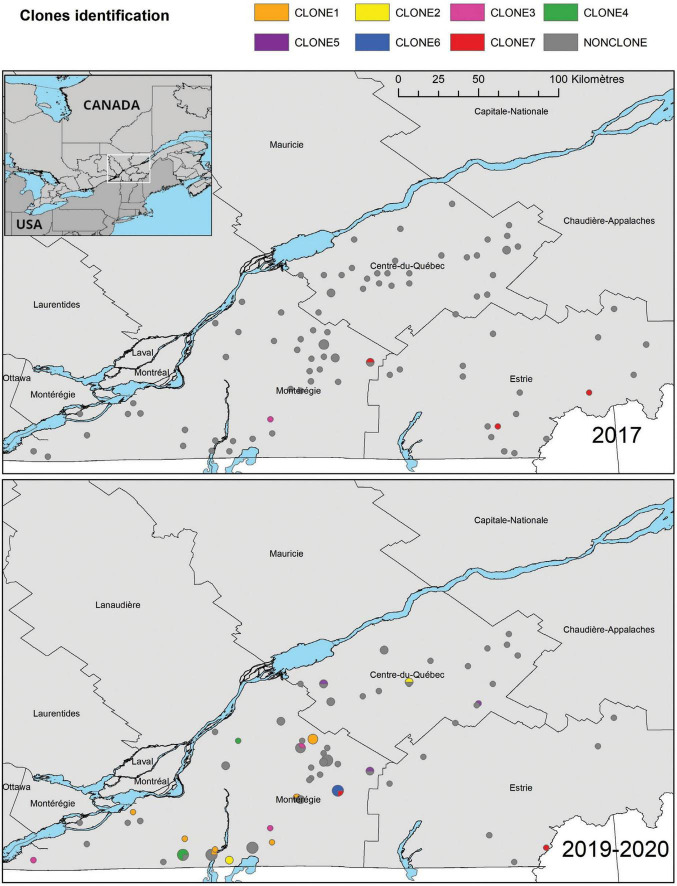
Geographical distribution of *E. coli* ESBL-AmpC isolates in the three study regions of the province of Québec, according to their sampling year. Clones were identified as a group of at least three isolates that differ by a maximum of 52 SNPs. A Lambert conformal conic projection (NAD 1983) was used for mapping.

Ten clonal lineages (sets of isolates belonging to the same cgMLST) were identified. The maximal number of SNPs used to define a clone using our definition was 52. With this definition, we identified 7 clones (illustrated with the maximum number of SNPs between two isolates in Figure 1). Distance matrix is provided in Supplementary Table 2. On the 31 isolates belonging to the 7 different clones, 20 (65%) were identified in calves’ samples, 8 (25%) were identified in manure pits and 3 (10%) were identified in cows’ samples. Isolates belonging to clone 6 (*n* = 3) were identified only on farm #95. Isolates belonging to other clones (1, 2, 3, 4, 5, 7) were identified across multiple farms (from 2 to 7), suggesting clonal dissemination within farms. As illustrated in Figure 2, clones 3 and 7 (in orange) were composed of isolates identified before and after the regulation implementation, suggesting the persistence of this clone over the 4-year period (from April 2017 to March 2021).

### 3.3 AMR profiles

All isolates were resistant to third generation cephalosporins, as they were selected following enrichment with cefotaxime. All but 4 isolates carried at least one gene or mutation among the following: *bla*_*SHV–*12_, *bla*_*CTX–M–*1_, *bla*_*CTX–M–*15_, *bla*_*CTX–M–*27_, *bla*_*CTX–M–*55_, *bla*_*CTX–M–*65_, *bla*_*CTX–M–*124_, *bla*_*CMY–*2_, and a mutation in the ampC promoter (42C). Using CARD (McArthur et al., 2013), we identified a determinant of efflux mechanism that might explain the cephalosporin resistance in the 4 other isolates. The AmpC promoter mutation was exclusively identified in ST88 isolates (data not shown).

**FIGURE 3:**
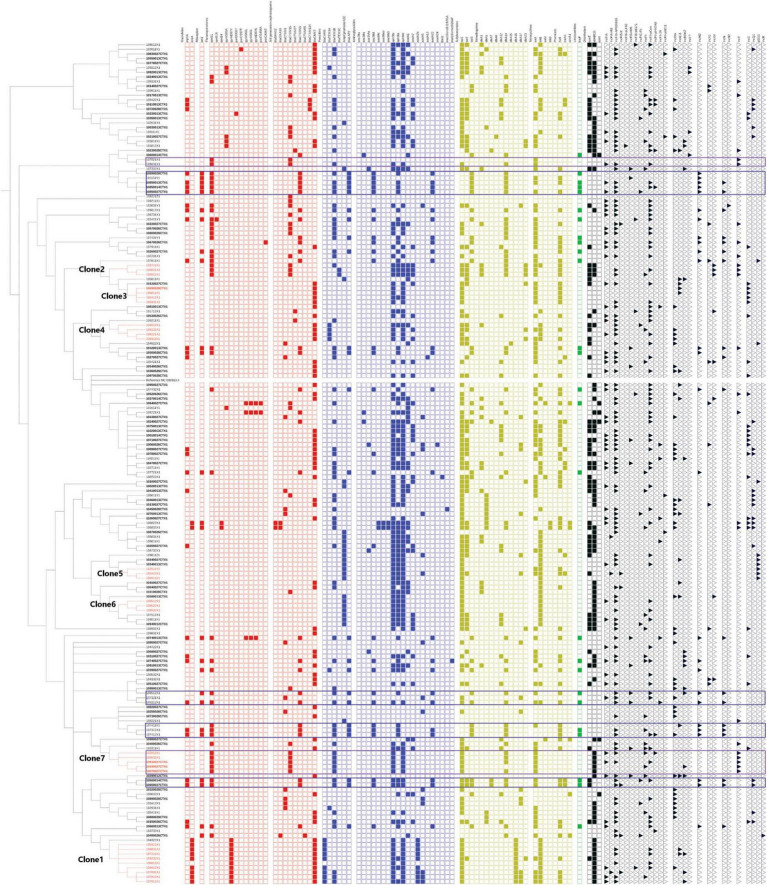
Overall phylogeny based on SNP distance of 182 ESBL *E. coli* isolates. The colored square indicates the presence of resistance genes. In red, critical high priority antimicrobials for human medicine. In blue, critical priority antimicrobials for human medicine. In yellow, high priority antimicrobials for human medicine. In green: antimicrobials important for human medicine. The importance of antimicrobials for human medicine was defined according to the World Health Organization. Black triangle indicates the presence of replicons. Violet frames aid the visualization of genes present when the replicon IncY is present. Blue frames aid the visualization of genes present when the replicon IncN and incHI2 are present. Samples with ID labels in bold indicate those collected prior to the implementation of the regulation.

Most identified resistance genes were similar between seasons (fall vs. spring) and between periods (pre- and post-regulation implementation). However, the *ereA* gene (*n* = 11) (responsible for macrolide resistance), the *bla_*SHV–*12_* gene (*n* = 2) (responsible for ESBL resistance), the *qnrB4* (*n* = 1) and *qnrB19* genes (*n* = 2) (responsible for fluoroquinolone resistance), the *aac(6′)-II-c* (*n* = 2), the *aac(6′)-Ib-cr* (*n* = 2), the *aac(6′)-Ib-3* (*n* = 2) and the *bleO* (*n* = 1) genes (responsible for aminoglycoside resistance), the *bla_*CARB*2_* (*n* = 10) and *bla_*TEM–*1*C*_* (*n* = 2) genes (responsible for penicillin resistance), the *tetD* gene (*n* = 2) (responsible for tetracycline resistance) and the *dfr15* (*n* = 1) and *dfr16* (*n* = 11) genes (responsible for trimethoprim resistance) were only identified post-regulation. On the other hand, the *aadA24* gene (*n* = 1), *bla_*CTX–M–*65_* gene (*n* = 1), the *drf7* (*n* = 4) and *drf8* genes (*n* = 1) were only detected pre-regulation. The details of genes identified in each isolate can be found in Figure 3.

### 3.4 Virulence profiles of interest

As illustrated in Figure 1, 16 isolates carried the 5 APEC-like predictors (*iss*, *iutA*, *ompT*, *hlyF*, *iroN*) (Johnson et al., 2008), commonly present in APEC isolates and, therefore, presenting a putative risk for poultry infection. They were mainly associated with the ST117 which belongs to predominant pathogenic APEC STs, and to ST58, ST88 which have also been reported as pathogenic STs. The 5 genes commonly present in ExPEC pathogen for human are *afa*, *sfa*, *iutA*, *pap*, *kpsMII* (Johnson et al., 2003). In our isolates, the gene *sfa* was not detected, however, the four other genes were detected. Isolates presenting the maximum number of these genes were associated with the ST10 and the ST2449.

One isolate (10990013CTX), belonging to the B1 phylogroup, O26:H11, MLST21 carried the *eae*, *espA*, *espB*, *espF*, and *tir* genes, therefore, was classified as an Enteropathogenic *E. coli* (EPEC) and was identified in a calf.

All isolates belonging to C (32/32), F (4/4) and G (8/8) phylogroups, all isolates but one belonging to the B1(56/57) phylogroup and 5/11 isolates belonging to phylogroup D (45%) carried the gene *lpfA*, encoding for the major fimbrial subunit of the log polar fimbriae (facilitating attachment) (Toma et al., 2006). Only two isolates carried *stx* toxins genes. These isolates were, respectively, O118:H2, ST17 belonging to B1 phylogroup and H32 (O was not identified) ST155 belonging to A phylogroup.

### 3.5 Plasmids carrying genes responsible for resistance to fluoroquinolones and 3rd generation cephalosporins

Our data strongly suggests that clone 7 carries a plasmid identified with the replicon IncY. It seems this plasmid carries the following resistance genes: *qnrS1*, *bla*_*CTX–M–*15_, *bla*_*TEM–*1_, *aph31b*, *aph61d*, *sul2*, *drf14*, *tetA*, and *sitABCD* (Figure 3, framed in violet), therefore carrying resistance to 6 families of antimicrobials, including 2 critical antimicrobials of category I (C3G and fluoroquinolones). Moreover, *qnrS1* and *bla*_*CTX–M–*15_ are found on the same contig on these isolates indicating with certainty that they are linked and flanked with the insertion sequence ISKpn19. We also noticed that no virulence genes seemed linked to the plasmid IncY.

Isolates carrying replicons IncHI2 and IncN seem to also carry resistance genes to 10 antimicrobial families, although we could not directly associate one or the other replicon with the different genes, because they were not found on the same contig. This replicon was identified pre- and post-regulation. Similarly, when identified together in one isolate, *bla*_*CTX–M–*55_ and *qnrs1* were not found on the same contig.

### 3.6 Clones of interest

Isolates belonging to clone 7 were identified pre- and post-regulation, in five different farms. Three out of five farms have bought cows within the last year at the time of the last sampling, and two were clients of the same veterinary clinic. However, no other link could be established between these farms. All isolates carried genes conferring resistance to 6 antimicrobial families and resistance to disinfectant and replicon IncY.

Isolates belonging to clone 1 were identified only post regulation, in 8 different farms, none of which were clients of the same veterinary clinic and two farms had bought cows within the past year at the time of the last sampling. Isolates all possessed a specific *gyrA* (D87Y) mutation found in no other isolate in the whole collection. They also carried genes conferring resistance to 6 other antimicrobial families (specifically *bla_*CMY–*2_*), however, no replicon could be identified clearly in these isolates (see Figure 2). Isolates from this clone carried the *mcmA* and the *papC* and *papA* genes and several genes involved in iron modulation.

Isolates belonging to clone 5 were identified only post-regulation, in 3 different farms, none of which were clients of the same veterinary clinic and one farm had bought cows within the last year at the time of the last sampling. Isolates carried the *ampC* promoter mutation and genes conferring resistance to 3 other antimicrobial families. The virulence profile of this clone was particular because its isolates carried several genes usually identified in the ExPEC pathotype (Johnson et al., 2008; Kathayat et al., 2021), being the *iss*, *iutA*, *ompT*, *fyuA*, *hra*, *ireA*, *irp2*, and *iucC* genes. These results suggest that isolates belonging to clone 3 might have the potential to cause infection in poultry.

Isolates belonging to clone 2 were identified only post-regulation in 2 different farms which were not clients of the same veterinary clinic and had not bought cows within the last year at the time of the last sampling. These isolates carried genes conferring resistance to 7 antimicrobial families and the replicon IncY and IncX. As they harbor the same resistance profile and the same replicons, these data suggest that the plasmid present in clone 1 is also present in clone 2.

Isolates belonging to clone 4 were identified only post-regulation and in only one farm. They carried only the *bla_*CMY–*2_* gene and an IncI replicon. The virulence profile of this clone was also of particular interest because these isolates carried several genes usually identified in the ExPEC (APEC-like) pathotype (Johnson et al., 2008; Kathayat et al., 2021), being the *afa*, *iutA*, *ompT*, *papC*, *papA*, *sitA*, *traT*, *cia*, *kpsE*, and *kpsM* genes. These results suggest that isolates belonging to clone 6 might have the potential to cause infection in both poultry and humans, as it carries 4 genes of the human ExPEC predictors (Johnson et al., 2003).

The geographical distribution of clones is illustrated in Figure 2. On this map, it is important to notice that clones 3 and 7 have been identified in 2017 and 2019-20, in different locations, suggesting they persisted through the 4-year period and disseminated in different farms. Clones 1, 2, 4, 5 and 6 were identified only in 2019-20. Moreover, clones 1 and 7 and have been identified all over the studied territory on farms over 100 km away.

## 4 Discussion

The main objective of this study was to characterize and assess the putative phylogenic link between ESBL/AmpC *E. coli* gathered on 87 dairy farms in Québec, over a 4-year period (pre- and post- regulation implementation). Our secondary objective was to determine the presence of plasmids carrying genes responsible for resistance to critical AM in these isolates, and to ascertain whether these plasmids were able to persist over a 4-year period. Indeed, deepening our knowledge on clonal and plasmid dissemination of resistance genes in the Québec cattle population would improve our ability to predict cross-resistance at the local level and provide data to improve biosecurity measures to limit dissemination.

Our analytical approach allowed us to examine the phylogenetic variation of ESBL/AmpC isolates from healthy cattle (calves, cows) and from their direct environment (manure pit). Firstly, the ESBL/AmpC isolates were very diverse. Indeed, all phylogroups were represented with a predominance of A and B1 which are the phylogroups commonly described in commensal *E. coli* in herbivores (Tenaillon et al., 2010). However, we also identified a variety of phylogroups (C to G) that are more commonly associated with pathogenic isolates (Denamur et al., 2021). We identified one EPEC (with the presence of *eae*) and several combinations of ExPEC genes, associated with known pathogenic STs. In terms of virulence, there were several sets of isolates of particular interest. Firstly, the 28 isolates belonging to ST88, phylogroup C, and presenting O8-O9 serogroups carried between 3 and 5 of the ExPEC predictors (Johnson et al., 2008) and were identified as putative pathogens in humans, domestic mammals and birds (Denamur et al., 2021). It is noteworthy that isolates of clone 3 belong to this group and carry resistance genes to 4 AM families. Secondly, 6 isolates belong to ST117 O137-O153/H4 that has been identified as a pathogen in birds (Denamur et al., 2021). Two isolates belong to phylogroup G, ST117, cgMLST 87147 and have been identified in calves. Isolates belonging to the same clonal lineage (same cgMLST) were identified causing omphalitis in Québec in poultry [unpublished data coming from the Animal Pathogenic and Zoonotic *E. coli* database (De Lagarde et al., 2020)]. Thirdly, the clone 6 belonged to the ST10 which has been associated with pathogenicity in Humans (Manges et al., 2019). Moreover, these latter isolates carry 4/5 genes predictors for human pathogenicity (Johnson et al., 2003) and thus, represent a putative risk for human health. Fourthly, we identified isolates belonging to the ST58 which seems to be an emergent uropathogenic *E. coli* (UPEC) in humans. However, our isolates did not carry the colV replicon associated with virulence (as it is commonly found in isolates of bovine origin) (Reid et al., 2022). Presence of ExPEC genes in these isolates is not surprising because they enhance survival by providing protection against predation by protozoa [such as amoebae (Alsam et al., 2006) and *Tetrahymena* spp. (Steinberg and Levin, 2007)]. However, our results highlight the importance of the surveillance of these strains in the bovine population due to their putative capacity to cross species barriers and cause disease in humans.

Although we observed a variety of strains, it is essential also to notice that we identified several clones. Our definition of a clone is based on several criteria, with a maximal number of SNPs (De Lagarde et al., 2021). This definition might be open to discussion because the mutation rate is subject to environmental pressure and is difficult to establish (Sniegowski et al., 1997; Reeves et al., 2011). Nevertheless, it is still a very stringent definition, and we found clonal isolates on different farms and persisting over a 4-year period. This result suggests that current biosecurity measures are not sufficient to limit the propagation of AMR genes from one farm to another, even though we were not able to determine the vector of dissemination in these farms. Several hypotheses can be considered. Firstly, wild birds have been proposed as a putative vector to spread clonal bacteria over a territory (Skarżyńska et al., 2021). However, it might not be the only route of dissemination. Another possibility may be dissemination through insects and especially through flies (Zurek and Ghosh, 2014). Although a fly usually covers a 2 miles diameter sector, some have been able to travel between 5 and 20 miles (Townsend, 1997). Moreover, it is also possible that they become trapped in vehicles and travel further. Other human vectors (such as animal transporters, inseminators, or other stakeholders) as well as other wildlife species have not been investigated in our study and represent a possible dissemination mode between farms. All these hypotheses are avenues for action to limit the spread of resistance genes and improve biosecurity in dairy farms in Québec.

It will be important to continue surveillance for clone 1, the only clone in our study that possessed a mutation (D87Y) in the *gyrA* gene. This mutation is known to confer resistance to nalidixic acid in *E. coli* isolates (Weigel et al., 1998). Indeed, clone 1 isolates were resistant to nalidixic acid but susceptible to fluoroquinolones (data not shown). This type of mutation might increase the fitness of the clone (Marcusson et al., 2009) and its capacity to spread efficiently. In addition, this clone was the most frequently observed in this study and it only appeared post-regulation, suggesting that it may harbor elements associated with evolution advantage. Moreover, isolates of this clone already possess virulence genes such as *papA* and *papC*, which are a part of the *pap* genes cluster encoding for the proteins required for P-fimbrial synthesis. The P fimbria is recognized as an essential adhesin in UPEC (Lane and Mobley, 2007). The acquisition of a plasmid carrying additional virulence or resistance genes might allow it to become more worrisome for bovine or human health.

The presence of *bla*_*CTX–M*–15_ and *qnrs1* on the same contig is quite alarming also because resistance genes to 3rd generation cephalosporins and fluoroquinolones are thus linked together. They confer, to isolates that carry them, resistance to 2 families of critical antimicrobials. Although short read data are limited to circularize plasmids, it is very likely that these two genes were carried by plasmids that carry also other resistance genes from other categories (possibly IncY). This type of plasmid has been detected over the world, in Nigeria, Africa (Alonso et al., 2017) and United Kingdom, Europe [Muna Anjum. abstract of the 9th symposium on antimicrobial resistance in animals and the environment (ARAE)]. This highlights their capacity of dissemination. Moreover, the co-resistance phenomenon enhances the importance of a judicious usage of all antimicrobials, and not only critical antimicrobial for human health. Indeed, through these putative plasmids, ESBL and fluoroquinolones genes might persist in the *E. coli* population even though their usage has stopped. The long-read sequencing of a few isolates of our collection would have been very instructive to document accurately these plasmids. It will be the subject of a subsequent study.

The *ampC* gene is located on *E. coli* chromosome and produces a class 1 cephalosporinase. In its normal state, the expression of *ampC* in wild-type cells is low and does not provide significant resistance to beta-lactam antimicrobials (Jacoby, 2009). However, various genetic changes can lead to increased expression of *ampC*, a condition known as *ampC* hyperexpression. In the 80s, *ampC* hyperproduction in *E. coli* was the main mechanism of resistance to third generation cephalosporins at that time. In recent years, with the emergence of plasmid-mediated *ampC*s (such as *bla*_*CMY*_), which are genes encoding for enzymes that confer resistance to a broader range of beta-lactam antimicrobials, *ampC* hyperproduction is no longer the dominant mechanism of resistance in *E. coli* (Findlay et al., 2020). However, recently it has regained interest in the scientific community as it has been identified in livestock and humans in the UK (Alzayn et al., 2020), in Netherlands (Ceccarelli et al., 2019), and in Belgium (Guerin et al., 2021). In several of these studies, the *ampC* hyperproduction mechanisms is due to the mutation −42 (C > T) and seems associated with the ST88. We identified similar phenomenon in our isolates in Québec Canada, which suggests that they might disseminate through clonal lineage around the world. This would need further investigation in a large international study.

It is interesting to note that most of the clonal isolates were sampled in calves. This information corroborates what had already been demonstrated previously (Horton et al., 2016; De Lagarde et al., 2022). To explain this, we can hypothesize that the immaturity of the calves’ microbiome is more prone to the persistence of bacteria and a fortiori *E. coli* clones with increased fitness. It seems also that feeding waste milk containing antimicrobial residues increase the number of resistant bacteria shed in feces in calves (Brunton et al., 2014). Another possibility is that calves receive more systemic oral treatment than cows, which tend to be treated locally, intravenously, or intramuscularly. Oral treatments are susceptible to modify the intestinal microbiome. Regardless of the reason, it means that calves should be manipulated more cautiously to limit the dissemination and the putative transmission of MDR clones to humans.

Our study presents limitations. Indeed, even if we gathered a lot of information’s in our questionnaires (Lardé et al., 2021), we still were not able to identify physical vectors for clones. In the future, it would be of interest to obtain data on animals’ movements. Flies and wild birds should also be considered in future samplings. These types of data should be gathered in the future to clarify the epidemiological link between farms which is essential to elaborate effective biosecurity measures to limit clonal spread. The other major limitation of our study, as already mentioned, is the lack of long read sequencing, which greatly limited our study and therefore our analysis on plasmid dissemination.

As a conclusion, we demonstrated that MDR ExPEC are present in the normal microbiota of cattle (more frequently in calves) and that AMR genes spread through farms. These genes can persist over a 4-year period in the dairy cattle population through both plasmids and *E. coli* clones despite important changes in AMU following the implementation of a new restricting regulation. In a previous paper, we demonstrated that the number of MDR isolates decreased between the two periods (De Lagarde et al., 2022). Taken together, these data demonstrate that, although efficient, the decrease in AMU is not enough to fight against AMR because gene dissemination is a complex phenomenon. Resistance gene surveillance should include the study of clones, their virulence, and their fitness. These data advocate changes to current AMR monitoring methods and highlight that biosecurity measures should be enhanced in this industry to limit this dissemination.

## Data availability statement

The datasets presented in this study can be found in online repositories. The names of the repository/repositories and accession number(s) can be found below: https://www.ncbi.nlm.nih.gov/, PRJNA716674; https://www.ncbi.nlm.nih.gov/, PRJNA1022465.

## Ethics statement

The animal studies were approved by the Animal Use Ethics and the Research Ethics Committees of the Université de Montréal. The studies were conducted in accordance with the local legislation and institutional requirements. Written informed consent was obtained from the owners for the participation of their animals in this study.

## Author contributions

ML: Conceptualization, Data curation, Formal analysis, Investigation, Methodology, Software, Visualization, Writing – original draft. JF: Conceptualization, Data curation, Formal analysis, Funding acquisition, Methodology, Supervision, Visualization, Writing – review & editing. MA: Conceptualization, Funding acquisition, Project administration, Supervision, Validation, Writing – review & editing. SD: Conceptualization, Funding acquisition, Investigation, Project administration, Resources, Supervision, Validation, Writing – review & editing. DF: Conceptualization, Funding acquisition, Investigation, Project administration, Supervision, Writing – review & editing. JM: Investigation, Methodology, Writing – review & editing. HL: Data curation, Investigation, Methodology, Writing – review & editing. CA: Funding acquisition, Project administration, Resources, Supervision, Writing – review & editing. M-EP: Funding acquisition, Project administration, Resources, Writing – review & editing. YT: Formal analysis, Writing – review & editing. J-PR: Formal analysis, Funding acquisition, Project administration, Resources, Supervision, Validation, Writing – review & editing.

## Footnotes

1
http://www.genomicepidemiology.org/

2
http://clermontyping.iame-research.center/

3
https://github.com/josephhughes/Sequence-manipulation/blob/master/Fasta2Phylip.pl

4
http://itol.embl.de

## Supplementary material

Supplementary Table 1 Quality data and phylogenetic characteristics.

Supplementary Table 2 Distance matrix.
